# A Rare Case of Epididymal Cyst Due to Schistosomiasis

**DOI:** 10.7759/cureus.5755

**Published:** 2019-09-25

**Authors:** Vikash Sinha, Manu Shankar, Nitin Sardana, Riti Aggarwal

**Affiliations:** 1 General Surgery, Fortis Escorts Hospital, Faridabad, IND; 2 Pathology, Fortis Escorts Hospital, Faridabad, IND

**Keywords:** schistosomiasis, epididymal cyst, carcinoma bladder

## Abstract

Schistosomal epididymitis is a very rare condition. Worldwide, very few cases have been reported, especially in India. Here is a case of schistosomal epididymitis that was found on histopathological examination of an epididymal cyst in a 32-year-old man in India. Patient presented with concerns of a right testicular swelling. Ultrasonography of scrotum showed an ill-defined echogenic lesion just above the head of right epididymis. Excision of epididymal cyst was performed. Histopathological examination showed eggs of schistosoma surrounded by abundant inflammatory infiltrate. Post-operatively, the patient was treated with single dose of praziquantel.

## Introduction

Schistosomiasis is a chronic infection caused by trematodes of genus Schistosoma. It is prevalent in the Middle East and African countries and is very rare in India. Schistosomiasis mainly infects the bladder and causes hematuria and squamous cell carcinoma of the bladder. Rarely does it involve the epididymis and testis. Here is a case of schistosomal epididymitis diagnosed after histopathological examination of a resected specimen.

## Case presentation

A 32-year-old man residing in Faridabad, India, presented with concerns of a painless swelling in his right scrotum for one week that was gradually increasing in size. He had no history of traveling abroad.

Physical examination revealed swelling of approximately 3 cm × 2 cm in the right epididymis. It was non-tender and cystic in consistency. Ultrasonography of the scrotum showed an ill-defined echogenic lesion of approximately 31 mm × 12 mm with a small, central hypoechoic area measuring approximately 7.5 mm × 5 mm in the right spermatic cord just above the level of the head of the right epididymis.

**Figure 1:**
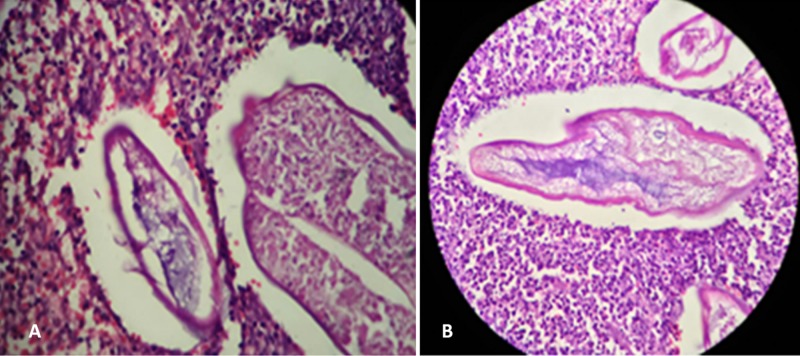
(A) A group of bilharzial ova within the interstitium of the epididymis(hematoxylin and eosin stain, x 40). (B) A higher power image of the same ova as (a) (hematoxylin and eosin stain, x 400).

After informed consent, excision of the epididymal cyst was performed. Histopathological examination showed fibroadipose tissue with eggs of Schistosoma surrounded by an abundant inflammatory infiltrate composed of foamy cells, eosinophils, and polymorphs (Figure 1).

The postoperative period was uneventful, and the patient was treated with a single dose of praziquantel.

## Discussion

Schistosomiasis of the urinary tract is endemic in the Middle East and African countries. This disease is acquired through infected water while bathing. The cercaria of the nematode Schistosoma hematobium penetrate the skin and reach the liver through blood vessels where they utilize erythrocytes and develop into male or female worms. The male forms a gynecophoric canal into which the female worm nestles. The worm has an affinity for the vesical venous plexus, which it reaches through the portosystemic anastomotic channels. In the bladder, the female worm enters a submucous venule where it lays approximately 20 eggs. These eggs have a terminal spine that penetrates blood vessels. The eggs are released through the urine. The ova, after reaching freshwater, rupture and form ciliated miracidium. These infect the intermediate host snail and form cercaria to complete the life cycle [1].

Acute schistosomiasis, also known as Katayama fever, presents with fever, lymphadenopathy, splenomegaly, eosinophilia, urticarial, and serum sickness-like disease. Chronic schistosomiasis presents with hematuria, terminal dysuria, early polypoidal lesions of the bladder obstructing the ureter and urethra, bladder ulcers, and squamous cell carcinoma of the bladder. It may also involve seminal vesicles and ejaculatory ducts. Involvement of these genitourinary structures may present with scrotal pain and a testicular mass. Sometimes, it also involves the epididymis, testis, uterus, vagina, ovaries, and fallopian tubes [2].

Only three cases of schistosomal epididymitis and 12 cases of testicular schistosomiasis have been reported worldwide [3-4]. In 2004, Alves, Assis, and Rezende reported a case of schistosomal epididymitis in a 32-year-old man. The patient presented with scrotal pain and a tender, hard, left epididymis. A left epididymectomy was done that showed eggs of Schistosoma mansoni [5].

Okani et al. reported a case of a 12-year-old boy with a left testicular mass. On exploration, a hard mass was found adherent to the epididymis. A biopsy revealed a chronic granulomatous inflammation around calcified Schistosoma hematobium eggs [6].

 Honare and Coleman reported a case of 54-year-old Canadian man with a painless swelling of his right testis. A biopsy showed schistosomiasis due to Schistosoma hematobium [3].

El-Hawary and Foda published five cases in 2016 in which they found Schistosoma eggs in the testis, prostate, and seminal vesicles [7].

Joshi reported a case of a 10-year-old boy with a slowly growing painless swelling of the right scrotum. On suspicion of a right-sided seminoma, a right orchidectomy was done. The biopsy showed ova of Schistosoma at different stages of degeneration and calcification [8].

 Al-Qahtani and Droupy reported a case of a 31-year-old man with primary infertility for nine years and a right-side testicular mass. Scrotal exploration and frozen sections showed schistosomiasis [4].

Praziquantel is the treatment of choice for schistosomiasis. A single dose of 40 mg/kg is an effective treatment. Other drugs that can be used are metrifonate, oxamniquine, and artemisinin-derivatives (artesunate and artemether).

## Conclusions

Schistosomiasis of the epididymis is a rare disease. It can present as an epididymal swelling that can be diagnosed after a histological examination of a resected specimen. Praziquantel is the treatment of choice.

## References

[1] Neal DE (2008). Bailey & Love’s short practice of surgery. NS, BulstrodeCJK, O’Connell PR. London: Hodder Arnold October.

[2] Ghoneim IA, Rabets JC, Mawhorter SD (2012). Tuberculosis and other opportunistic infections of the genitourinary system. Campbell-Walsh Urology.

[3] Honare L, Coleman G (1975). Solitary epididymal cyst. Can J Surg.

[4] Al-Qahtani SM, Droupy SJ (2010). Testicular schistosomiasis. Saudi Med J.

[5] Alves LS, Assis BPS, Rezende MMB (2004). Schistosomal epididymitis. Int Braz J Urol.

[6] Okani CO, Nyaga T, Otene BS, Uji FO, Ngbea JA (2014). A case report on a 12-year-old male with left epididymal swelling secondary to schistosomiasis. Pathology.

[7] El-Hawary AK, Foda AA-RM (2016). Incidentally detected schistosomiasis in male genital organs: case reports and review of literature. Am J Cancer Case Rep.

[8] Joshi RA (1967). Total granulomatous infarction of testis due to Schistosoma haematobium. J Clin Pathol.

